# Twenty-Four-Hour Movement Behaviors and Adult Mental Health: A Bibliometric Analysis of Global Trends, Research Hotspots, and Emerging Directions (2016–2026)

**DOI:** 10.3390/healthcare14213576

**Published:** 2026-10-22

**Authors:** Lifeng Yao, Jinxia Jiang, Xianliang Liu, Xia Duan

**Affiliations:** 1 Nursing Department, Shanghai Key Laboratory of Maternal Fetal Medicine, Shanghai Institute of Maternal-Fetal Medicine and Gynecologic Oncology, Shanghai First Maternity and Infant Hospital, School of Medicine, Tongji University, Shanghai 200092, China; 2 Department of Emergency, Shanghai Tenth People’s Hospital, School of Medicine, Tongji University, Shanghai 200072, China; 3 School of Nursing and Health Sciences, Hong Kong Metropolitan University, Hong Kong, China

**Keywords:** 24-hour movement behaviors, physical activity, sedentary behavior, sleep, mental health, bibliometric analysis

## Abstract

Background: Physical activity, sedentary behavior, and sleep constitute interdependent components of the 24-hour day and are jointly associated with mental health in adults. This study aimed to examine the global research landscape, hotspots, and emerging trends in this field from 2016 to 2026. Methods: Records published between 1 January 2016,and 30 April 2026, were retrieved from the Web of Science Core Collection. The bibliometrix package (version 5.3.0), implemented in R (version 4.5.2), was used to analyze publication and citation trends and contributions from countries, institutions, authors, and journals. Country-level publication output was quantified using full counting of unique document–country pairs, such that each publication was counted only once for a given country, whereas internationally co-authored publications were credited to all participating countries. Country citation rankings were determined by aggregating citations according to the corresponding author’s country of affiliation. VOSviewer (version 1.6.20) was used to construct country-level co-authorship networks using full counting and to generate keyword co-occurrence networks. The R packages ggplot2 and circlize were used for additional visualizations, including chord diagrams of citation relationships among journals. Results: A total of 527 publications were included. The annual global production from 2016 to 2026 reflected an overall upward trend with significant fluctuations. The amount of global output peaked in 2024 with 147 publications, whereas the number of total citations reached its highest in 2022 (2702). The United States was involved in 152 of the included publications, China in 118, and Australia in 87. Australia recorded the highest total citations (1757), followed by the United States (1667) and China (1556). Keyword overlay visualization revealed a shift from traditional behavioral epidemiology focused on workplace behaviors and subjective well-being toward integrated, precision-oriented mental health research emphasizing aging, cognitive health, circadian rhythm, and objective behavioral monitoring. Emerging hotspots included machine learning, compositional data analysis, isotemporal substitution, and accelerometer-based assessment. Conclusions: Research on 24-hour movement behaviors and adult mental health has evolved into an increasingly interdisciplinary field, with growing emphasis on integrated behavioral approaches, objective assessment methods, and healthy aging.

## 1. Introduction

Adult mental health has become an important public health issue worldwide, with depression and anxiety contributing substantially to disability and healthcare burden [1]. More than 440 million people are globally affected by mental disorders, and these conditions are associated with poorer quality of life and reduced social and occupational functioning [2,3]. A Global Burden of Disease (GBD) study showed that mental disorders are among the leading causes of years lived with disability, underscoring the need for effective mental health promotion and targeted interventions [2].

In recent years, increasing attention has been directed toward the role of lifestyle behaviors in mental health. Specifically, maintaining adequate physical activity [4], reducing sedentary time [5,6], and ensuring moderate sleep duration [7] alongside high sleep quality [8] have each been linked to better mental health. Physical activity, sedentary behavior, and sleep are intrinsically co-dependent within a finite 24-hour window, and altering one behavior inevitably shifts the others [9,10]. Consequently, researchers have increasingly adopted the “24-hour movement behaviors” framework, which considers these behaviors collectively rather than independently in relation to health outcomes. This holistic perspective is reflected in major public health guidelines, such as Canada’s integrated 24-Hour Movement Guidelines across the lifespan, which jointly specify recommended amounts of physical activity, limited sedentary time, and sufficient sleep for “a healthy day” [11].

Previous studies have confirmed the interactive benefits among these behaviors [12]. For instance, high physical activity combined with good sleep quality was linked to the lowest levels of depression and anxiety in older adults [13]. Among young adults, replacing sedentary time with moderate or vigorous activity reduced the odds of anxiety and depressive symptoms in a distinct dose–response pattern [5]. Several reviews have examined the relationship between 24-hour movement behaviors and mental health [14,15]. However, studies have examined a wide range of mental health outcomes, including depression, anxiety, stress, loneliness, and happiness, which limits direct comparison between findings [14,15,16]. Furthermore, the rapid growth of publications in this field has made it increasingly difficult to track evolving research themes and identify emerging areas of interest. Despite the expanding literature, comprehensive bibliometric analyses remain limited, making it challenging to identify current knowledge gaps and future research priorities.

Further synthesis of the literature is therefore needed. To our knowledge, no bibliometric study has specifically examined research on 24-h movement behaviors and mental health. Therefore, the present study addresses this gap by conducting a comprehensive bibliometric analysis of publications over the past decade, with the aim of characterizing the structure and development of this research field. The aims of this study were to: (i) characterize global patterns of research output, (ii) identify leading countries, institutions, journals, and authors, (iii) explore thematic hotspots and emerging trends, and (iv) assess the role of 24-h movement behaviors within mental health research.

## 2. Materials and Methods

### 2.1. Study Design

This study employed a bibliometric analysis to explore the landscape of 24-hour movement behaviors and mental health between 1 January 2016 and 30 April 2026. Bibliometric analysis provides a quantitative approach to examine published literature, identifying influential studies, as well as patterns across authors, journals, institutions, and countries [17,18]. Combined with citation analysis, which assesses the academic impact and visibility of publications [19,20], bibliometric approaches can help clarify current research trends and inform future directions.

### 2.2. Data Collection

Documents were retrieved from the Web of Science Core Collection on 30 April 2026. We used the following strategy: TS = (“24-hour movement behaviours*” OR “compositional analys*”) AND TS = (“mental health” OR “psychosocial” OR “psychological” OR “wellbeing” OR “well-being” OR “social health” OR “social emotional”); additional details are provided in Supplement File S1.

### 2.3. Eligibility Criteria

The inclusion criteria for eligible publications were as follows: (1) studies published between 1 January 2016 and 30 April 2026; (2) studies focusing on 24-hour movement behaviors, including physical activity, sedentary behavior, and sleep; (3) studies investigating mental health-related outcomes, including psychological, psychosocial, social-emotional, and well-being-related domains; and (4) studies published in English.

The exclusion criteria were as follows: (1) duplicate records retrieved from the database; (2) studies involving children or adolescents; (3) document types including early access, meeting abstracts, editorial materials, proceeding papers, corrections, and letters; and (4) studies not relevant to the relationship between 24-hour movement behaviors and mental health after title and abstract screening.

**Figure 1:**
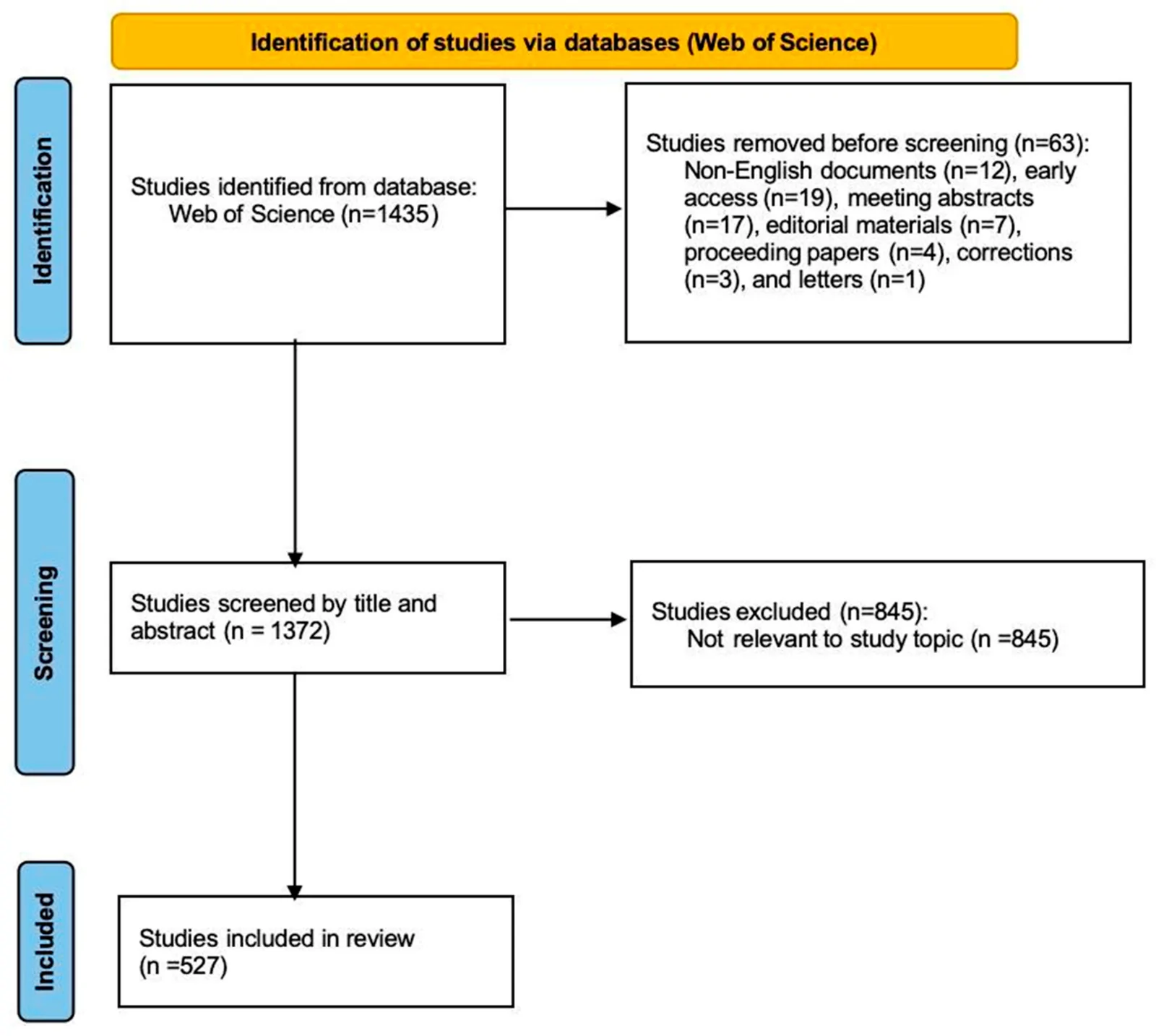
Flowchart of data collection process.

A total of 1435 documents were initially identified from the Web of Science Core Collection on 30 April 2026, using the predefined search strategy. After screening and eligibility assessment, 527 publications were included in the final analysis (Figure 1).

### 2.4. Data Analysis and Visualization

In this study, VOSviewer (version 1.6.20; Centre for Science and Technology Studies, Leiden University, Leiden, the Netherlands) and the bibliometrix package (version 5.3.0; K-Synth Srl, Naples, Italy), implemented in R (version 4.5.2; R Foundation for Statistical Computing, Vienna, Austria), were used for data mining and visualization. VOSviewer, a Java-based tool, enables the handling and visualization of large-scale bibliometric networks [21], while bibliometrix provides a comprehensive framework for the systematic analysis of bibliographic data within R [22].

VOSviewer was used to construct co-authorship and keyword co-occurrence networks using the full-counting method, with the default association-strength normalization applied to network layout and clustering. Bibliometrix was used to extract data on publication output, collaboration patterns, sources, and keywords.

Country-level publication output was calculated using full counting, with each unique document–country pair treated as a single counting unit. Each publication was counted no more than once for a given country, irrespective of the number of authors or affiliations associated with that country, whereas internationally co-authored publications were counted once for each participating country. For country-level citation analysis, publications and their citations were attributed to the corresponding author’s country of affiliation, and countries were ranked according to the total citations received by their attributed publications. The country-level co-authorship network was constructed in VOSviewer using full counting, with country names retained as parsed by VOSviewer from the Web of Science affiliation data.

In the initial screening stage, two independent reviewers evaluated titles and abstracts according to the predefined inclusion criteria. Records were subsequently exported from the Web of Science Core Collection in .txt format, and duplicate records were removed because some publications had been indexed multiple times within the database. The final dataset was analyzed using VOSviewer (version 1.6.20) and the bibliometrix package (version 5.3.0) in R (version 4.5.2), together with ggplot2 (version 4.0.2; Posit Software, PBC, Boston, MA, USA), for bibliometric analysis and data visualization. For additional journal network visualization, data were exported from the bibliometrix platform and visualized in VOSviewer. The resulting map and network files were subsequently imported into R and visualized using the circlize package (version 0.4.18; Zuguang Gu, Shenzhen University of Advanced Technology, Shenzhen, China) to generate chord-style representations of journal relationships. Additional Excel-based data processing and organization were performed using original datasets exported from the bibliometrix platform.

## 3. Results

### 3.1. Analysis of Global Trends

**Figure 2:**
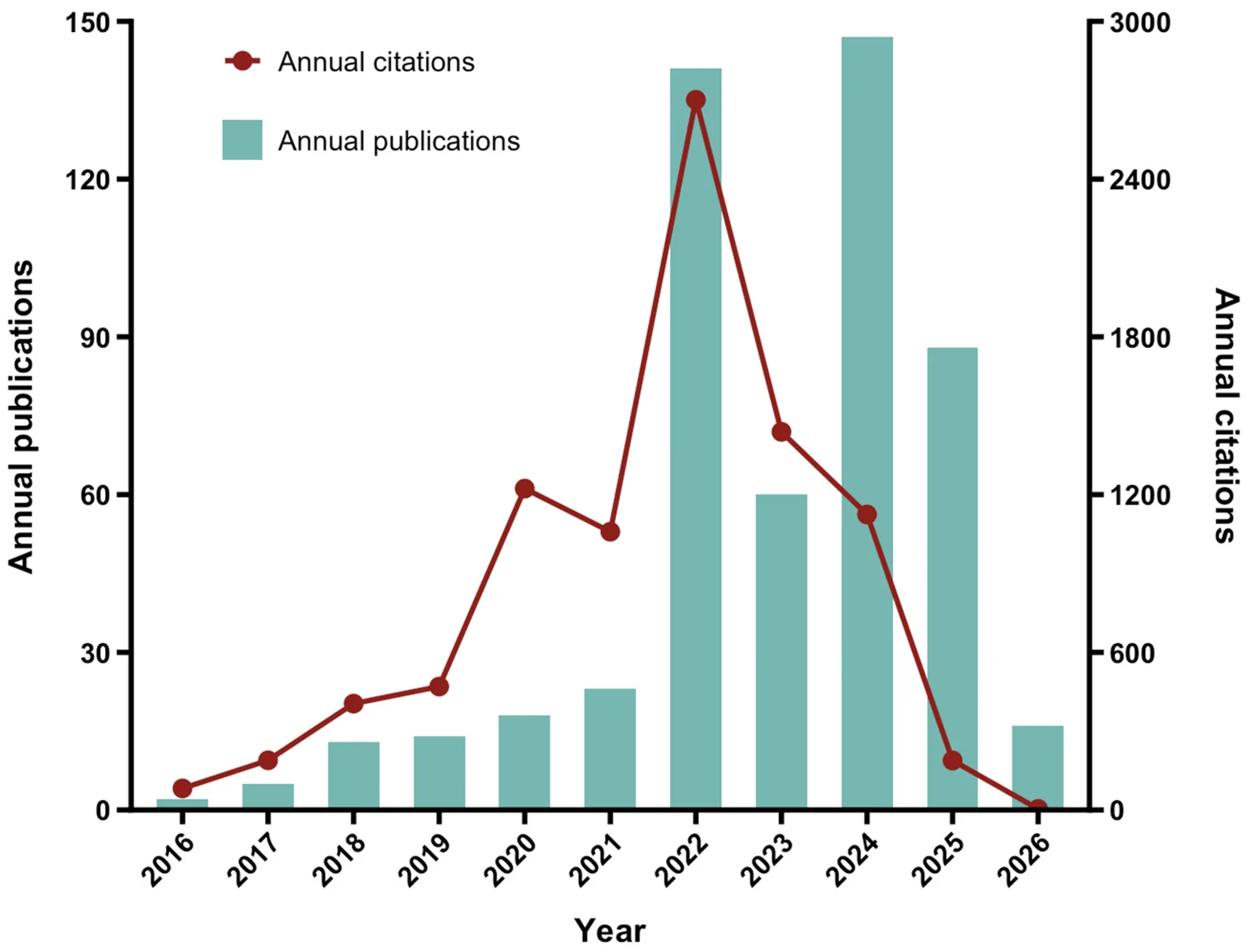
Global trends of publication and citation during 2016–2026.

A total of 527 documents were identified after data screening procedures. As illustrated in Figure 2, the annual global production from 2016 to 2026 reflected an overall upward trend with significant fluctuations. The amount of global output peaked in 2024 with 147 publications, whereas the number of total citations reached its highest in 2022 at approximately 2702.

**Figure 3:**
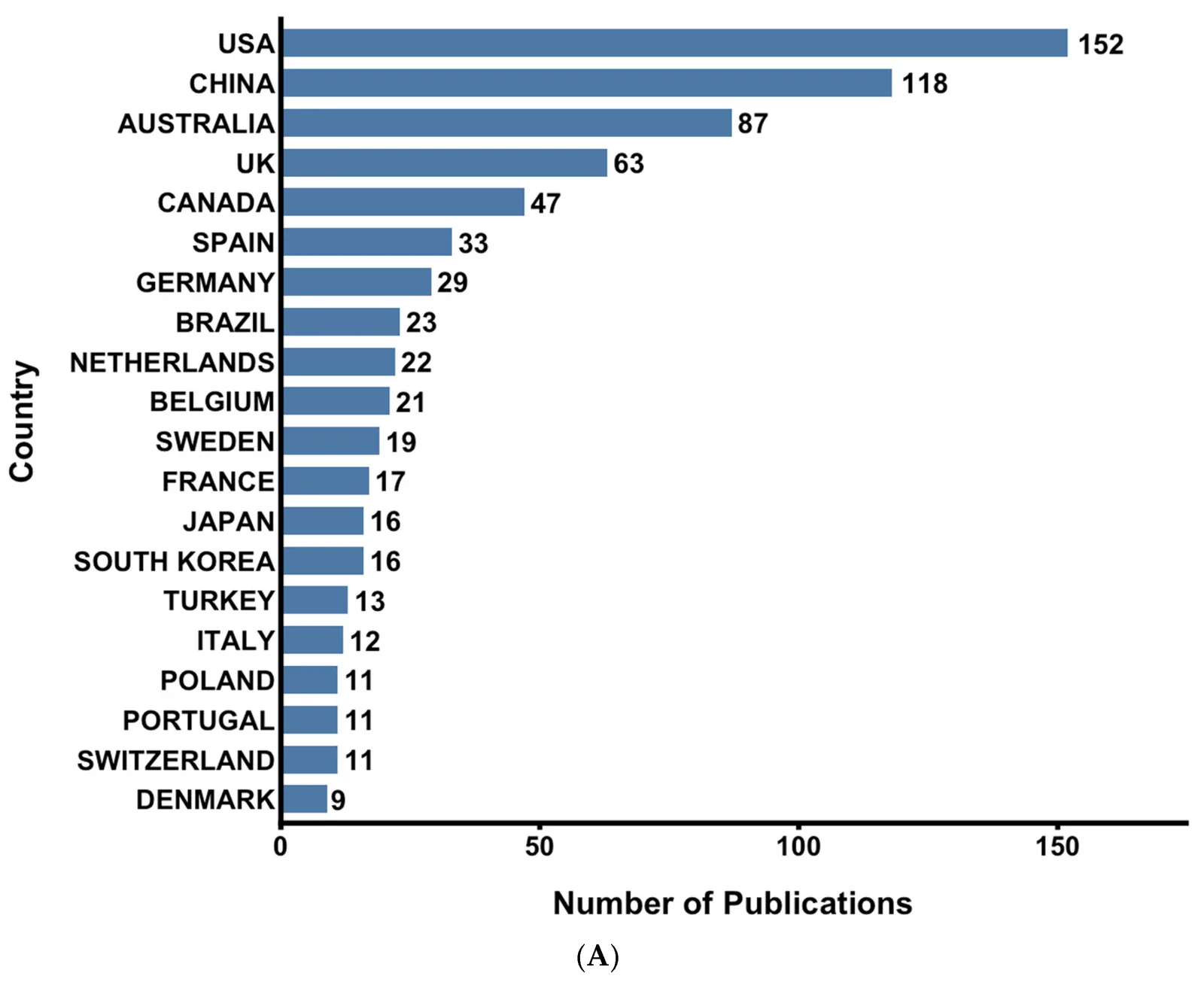

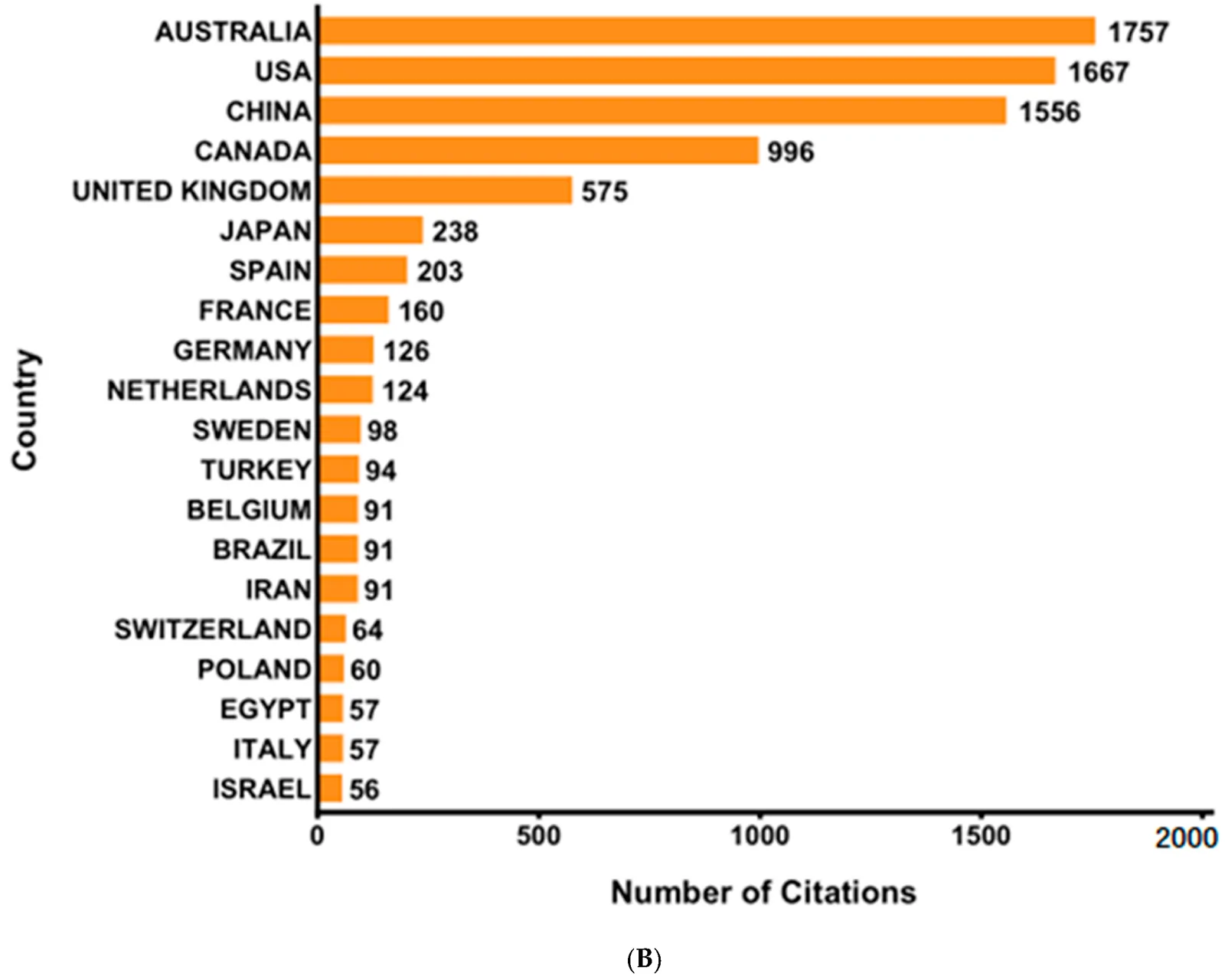

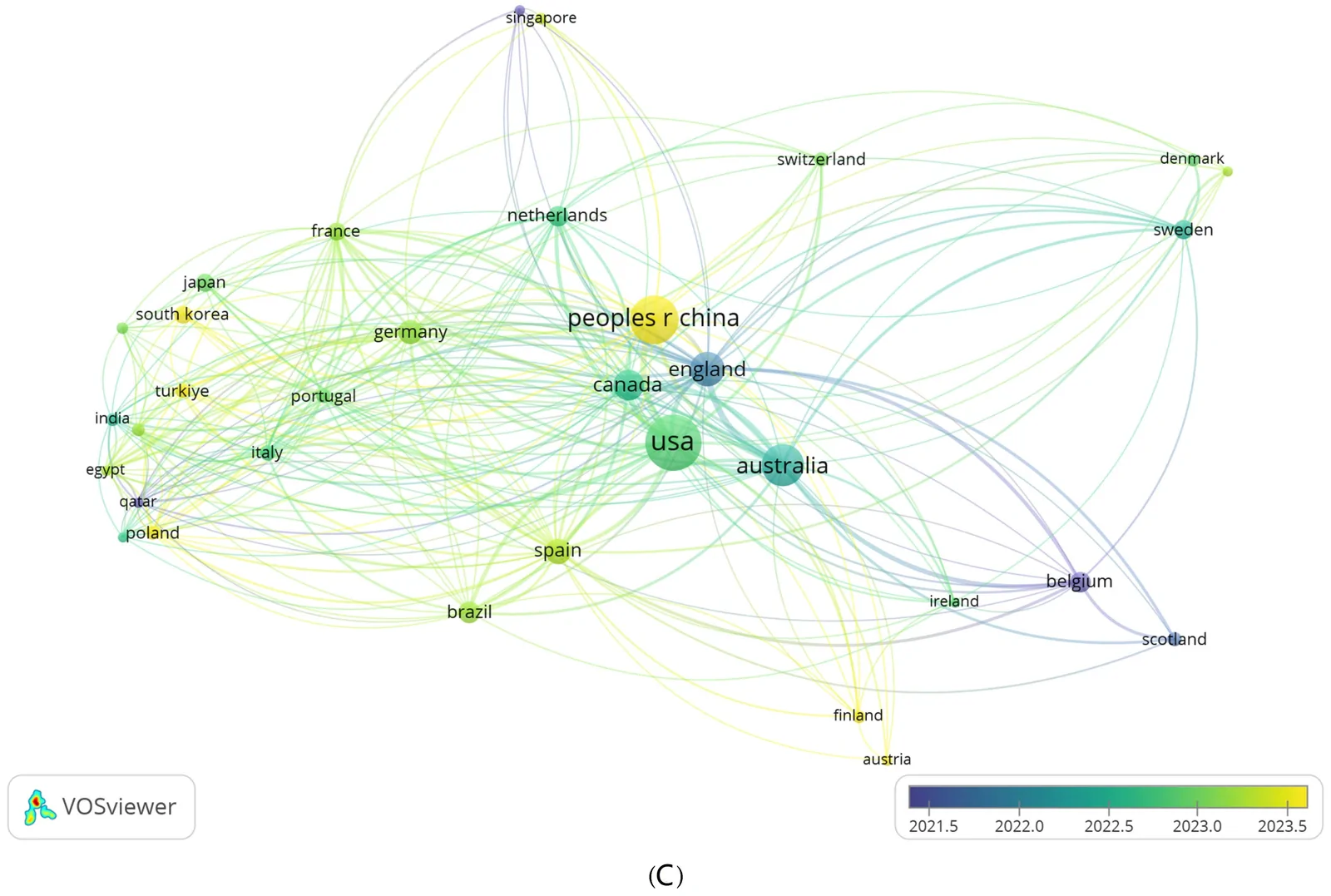
Country-level publication output and citation impact. (**A**) Top 20 countries by publication output, based on full counting of unique document–country pairs. (**B**) Top 20 countries by total citations, based on the corresponding author’s country of affiliation. (**C**) Country co-authorship overlay network.

A total of 72 countries/regions were identified in this study. The United States had the highest publication output (*n* = 152), followed by China (*n* = 118) and Australia (*n* = 87) (Figure 3A). Figure 3B shows that the top three countries in total citations were Australia (*n* = 1757), the USA (*n* = 1667), and China (*n* = 1556). Figure 3C displays the temporal visualization for country co-authorship networks. The size and color of the node reflect the academic output and activity of a country, while the strength of collaborations between countries is indicated by the thickness of the connecting line.

### 3.2. Analysis of Institutions and Authors

**Figure 4:**
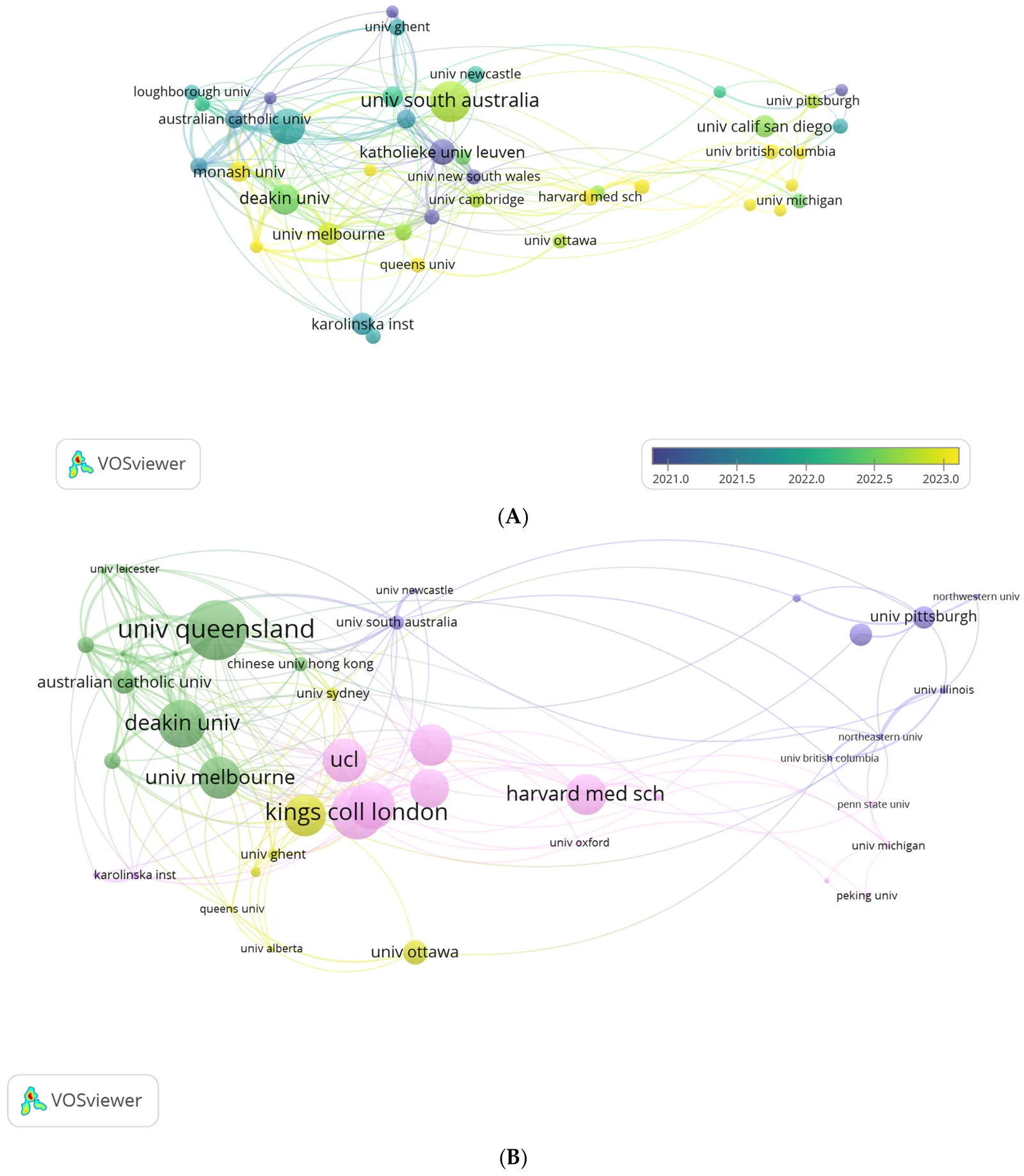
Institutional co-authorship networks of (**A**) temporal evolution and (**B**) citation.

There were 993 organizations that participated in the research of this field. Harvard University was the most productive institution (*n* = 36), followed by Adelaide University (*n* = 35) and the University of California System (*n* = 31) (Table 1). The temporal evolution of institutional output is represented in Figure 4A. The node size represents the output of an institution, while the color reflects the chronological trend of academic activity. The institutional co-authorship networks in citations are presented in Figure 4B, and the node size reflects the total citations of an institution, while the node color denotes the collaborative cluster. A denser concentration of nodes and edges within a cluster indicates a stronger collaborative relationship among those institutions.

**Figure 5:**
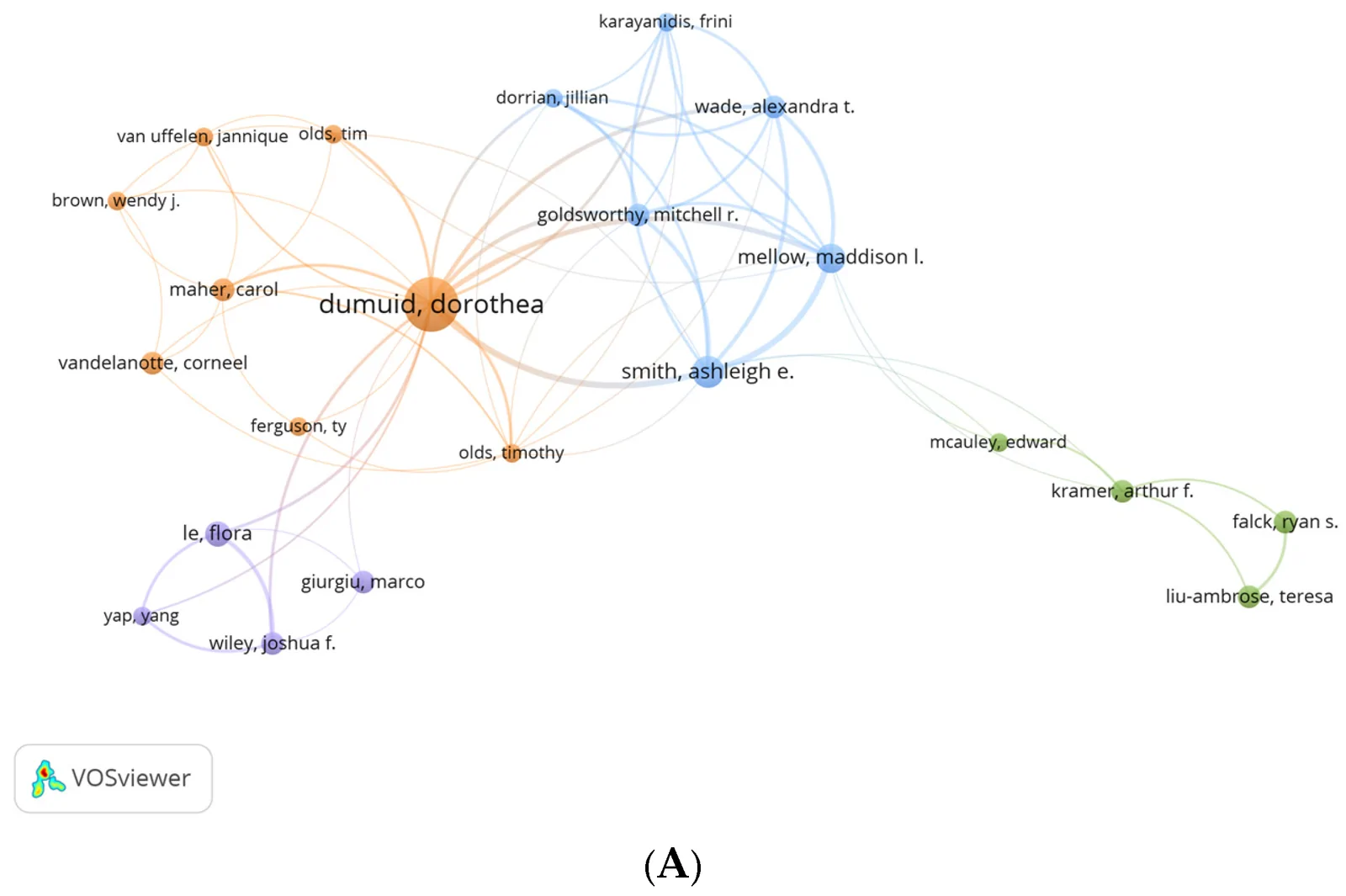

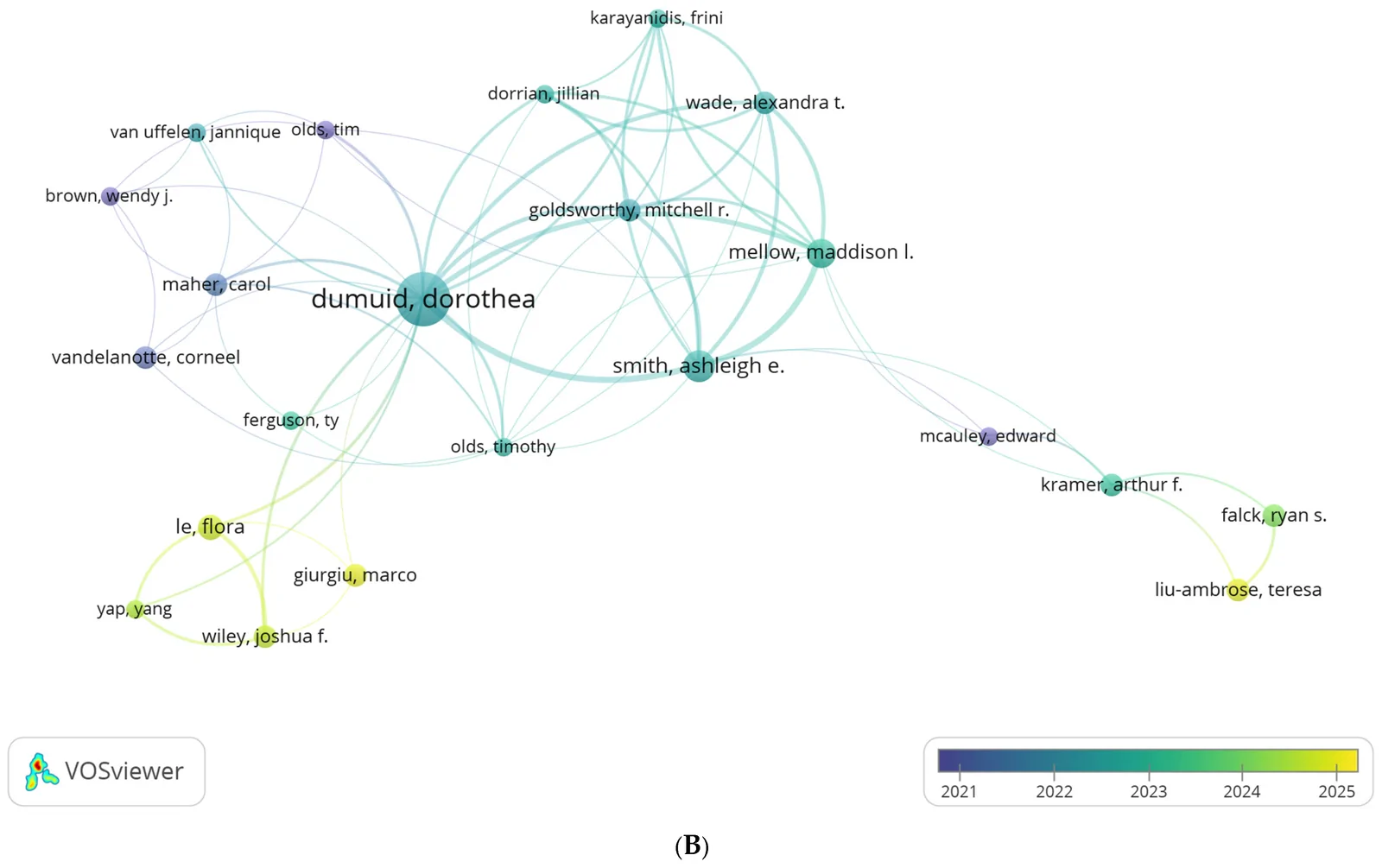
(**A**) Author co-authorship networks of clusters. (**B**) Author co-authorship networks of temporal evolution.

In this study, a total of 3301 author name records were identified in the retrieved publications.. D. Dumuid was the most productive author (*n* = 15), followed by L. Li (*n* = 8) and A. E. Smith (*n* = 7) (Table 2). On the other hand, the top three most cited authors were B. Stubbs (*n* = 1143), D. Vancampfort (*n* = 921), and J. Firth (*n* = 907) (Table 2). The collaborative clusters among authors are displayed in Figure 5A, and the size of the node reflects the scientific production of an author. Figure 5B represents the chronological pattern of co-authorship networks, and the color scale indicates the active period of an author.

### 3.3. Analysis of Journals and References

The impact factor and journal citation reports are common indicators applied to measure the academic impact of journals [23]. In our present study, we detected 285 journals and 8884 citations in total. Table 3 lists the leading journals in publications. The top three most productive journals were BMC Public Health (*n* = 31, IF = 3.6, Q1), International Journal of Environmental Research and Public Health (*n* = 18), and Journal of Affective Disorders (*n* = 16, IF = 4.9, Q1). According to the Journal Citation Reports 2025, seven of the top ten most productive journals were distributed in the Q1 region. International Journal of Behavioral Nutrition and Physical Activity ranked first in terms of impact factor among these top journals, with the highest impact factor of 5.5. As shown in Table 4, World Psychiatry (*n* = 784) had the highest citation, followed by Nature Reviews Endocrinology (*n* = 474) and Proceedings of the National Academy of Sciences of the United States of America (*n* = 428). All of the top ten most cited journals were Q1 journals, and World Psychiatry had the greatest impact factor of 65.8. 

In bibliometrics, local and global citations differentiate a publication’s academic impact within its immediate field from its broader cross-disciplinary reach [24,25]. In this study, we extracted a total of 25,413 relevant references. The publication in Journal of Science and Medicine in Sport by Wei, Jingkai et al. had the highest local citation of 17 (Table 5), while the manuscript published in World Psychiatry by Firth, Joseph et al. had the greatest global citation of 784 (Table 6).

**Figure 6:**
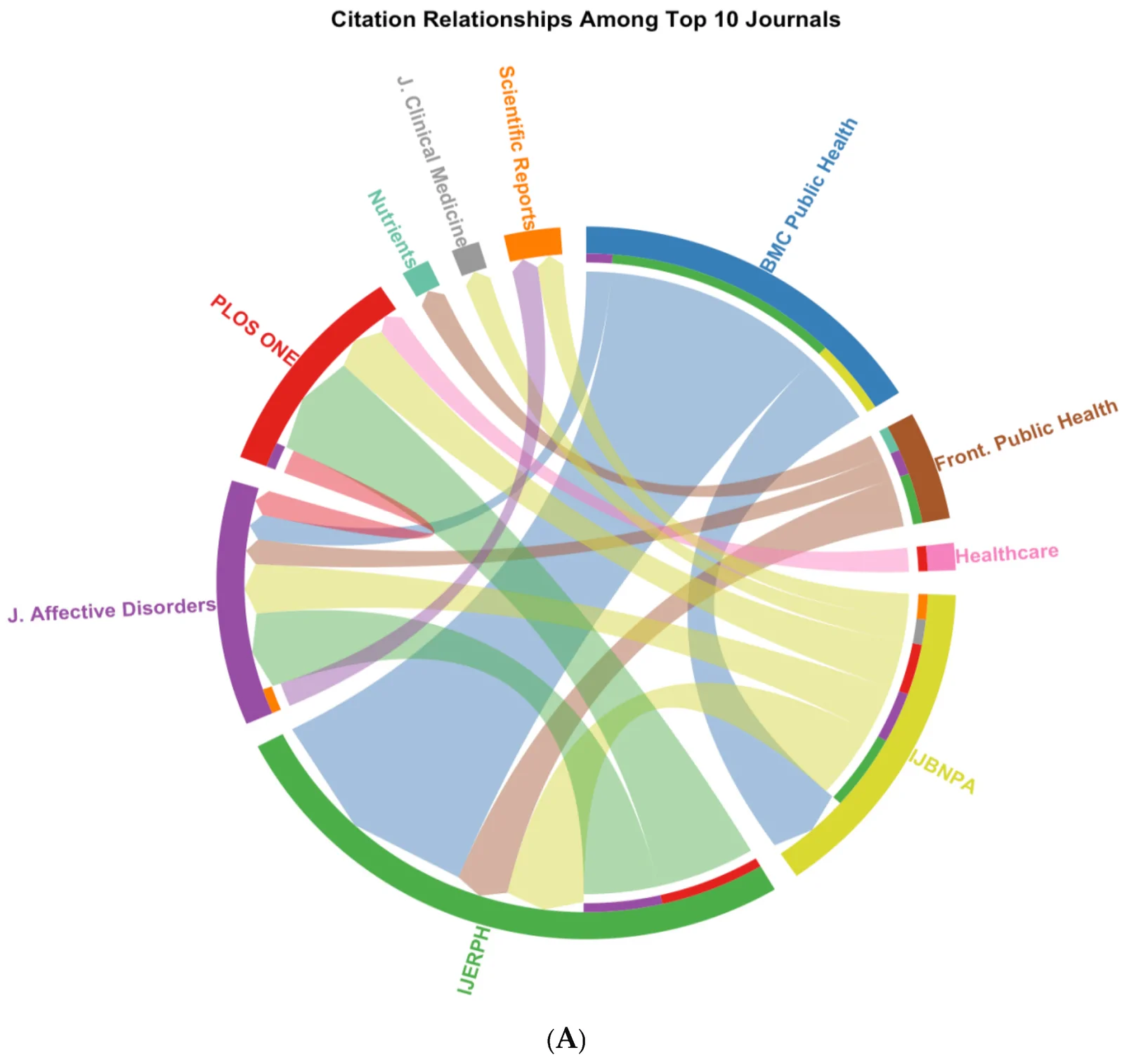

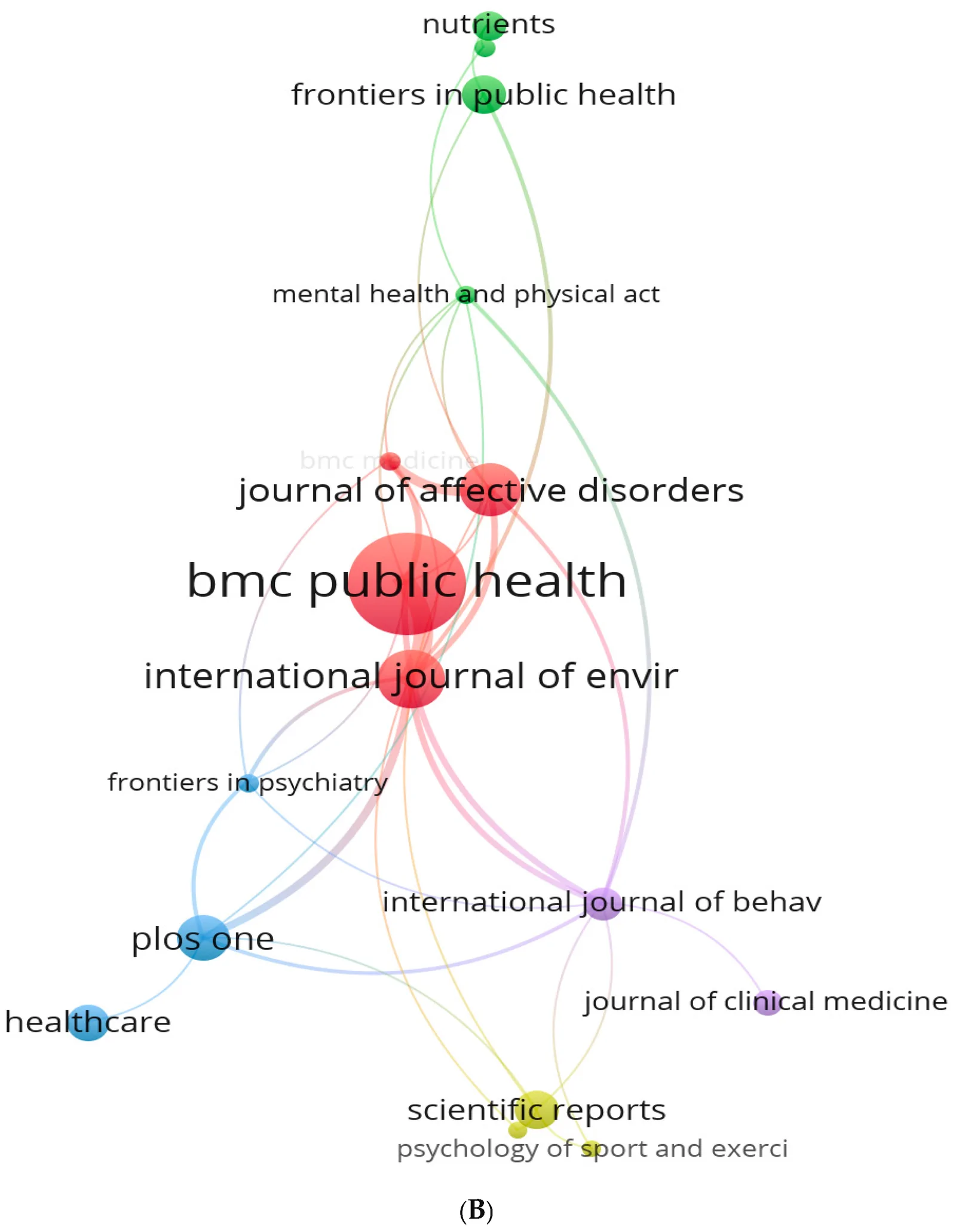
(**A**) Citation network analysis of publications. (**B**) Journal co-citation network.

Citation network analysis revealed dense inter-journal connections among the top 10 journals (Figure 6A).

*International Journal of Environmental Research and Public Health* (IJERPH) and *Journal of Affective Disorders (J. Affective Disorders)* were the most frequently cited journals within the network, whereas *International Journal of Behavioral Nutrition and Physical Activity* (IJBNPA) and *BMC Public Health* exhibited the highest levels of citing activity.

The journal co-citation network showed a core–periphery pattern with five clusters (Figure 6B). The red cluster occupied the center of the network and included *BMC Public Health*, the *Journal of Affective Disorders*, the *International Journal of Environmental Research and Public Health* (IJERPH), and *BMC Medicine*. *BMC Public Health* was represented by the largest node, followed by the *Journal of Affective Disorders* and IJERPH. The green cluster included *Nutrients*, *Frontiers in Public Health*, and *Mental Health and Physical Activity*, while the blue cluster comprised *PLoS One*, *Healthcare*, and *Frontiers in Psychiatry*. The purple cluster consisted mainly of the *International Journal of Behavioral Nutrition and Physical Activity* and the *Journal of Clinical Medicine*, and the yellow cluster included *Scientific Reports* and *Psychology of Sport and Exercise*. Co-citation links were observed both within and across clusters.

### 3.4. Analysis of Keywords and Hotspots

**Figure 7:**
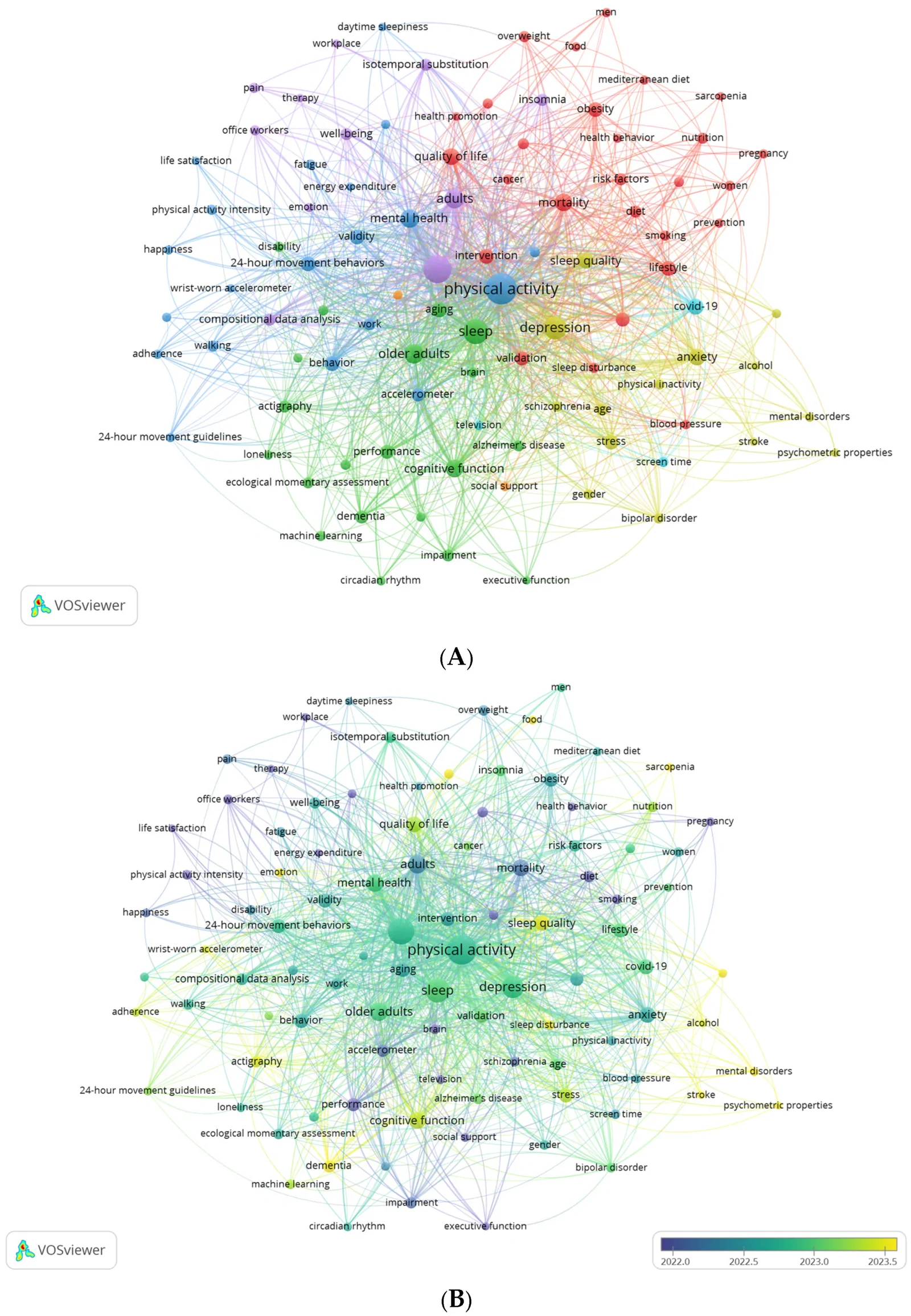
Keywords co-occurrence networks of (**A**) clusters and (**B**) temporal evolution.

In this study, a total of 2441 keywords were collected and grouped into five co-occurrence clusters. As demonstrated in Figure 7A, the red cluster primarily included keywords related to lifestyle and physical health, such as “cancer”, “blood pressure” “diet”, “food”, “health behavior”, “health promotion”, “lifestyle”, “mediterranean diet”, “mortality”, “nutrition”, “obesity”, “overweight”, “intervention”, “men”, “sleep disturbance”“pregnancy”, “prevention”, “quality of life”, “risk factors”, “sarcopenia”, “smoking”, “validation”and “women”. The green cluster featured keywords of “actigraphy”, “aging”, “alzheimer’s disease”, “brain”, “circadian rhythm”, “cognitive function”, “dementia”, “disability”, “ecological momentary assessment”, “executive function”, “impairment”, “loneliness”, “machine learning”, “older adults”, “performance”, and “sleep”. The yellow cluster encompassed keywords like “age”, “alcohol”, “anxiety”, “bipolar disorder”, “depression”, “gender”, “mental disorders”, “physical inactivity”, “psychometric properties”, “schizophrenia”, “sleep quality”, “stress”, and “stroke”. Furthermore, the blue cluster consisted of keywords including “24-hour movement behaviors”, “24-hour movement guidelines”, “accelerometer”, “adherence”, “behavior”, “daytime sleepiness”, “energy expenditure”, “fatigue”, “happiness”, “life satisfaction”, “mental health”, “physical activity”, “physical activity intensity”, “television”, “covid-19”, “screen time”, “validity”, “walking”, “work”, and “wrist-worn accelerometer”. Finally, the purple cluster involved “adults”, “compositional data analysis”, “isotemporal substitution”, “office workers”, “workplace”, “pain”, “therapy”, “emotion”, "insomnia” and “well-being”.

As shown in Figure 7B, which exhibits the chronological evolution of the keywords, a distinct shift in research focus was observed. The earlier hotspots predominantly included traditional risk factors such as “diet”, “smoking”, and “mortality”. Over time, the hotspots gradually shifted towards more specific assessment and cognitive domains, highlighting terms like “24-hour movement behaviors”, “24-hour movement guidelines”, “actigraphy”, “dementia”, “cognitive function”, and “mental disorders”.

## 4. Discussion

Although previous bibliometric studies have examined 24-hour movement guidelines in children and adolescents [43], this study is the first to provide a systematic mapping of the integrated literature on 24-hour movement behaviors and mental health in adults. The present study offers three notable contributions: (1) an exclusive focus on adult populations rather than children and adolescents; (2) a broader and more comprehensive inclusion of mental health-related domains and subfields; and (3) the identification of promising directions for interdisciplinary collaboration and clinical translation.

### 4.1. General Trends

Publication and citation activity in this field expanded substantially from 2016 on-ward, with local publication peaks in 2022 and 2024, and a notable citation peak in 2022. To contextualize these trends, the study period can be divided into three phases: the pre-pandemic period (2016–2019), the pandemic period (2020–2022), and the late- and post-pandemic period (2023–April 2026).

Lockdown and social-distancing measures implemented during the pandemic substantially altered daily movement patterns. A systematic review found that physical activity generally decreased and sedentary behavior increased during lockdowns [44], while another systematic review and meta-analysis reported reductions in moderate-to-vigorous physical activity and sleep quality, together with an increase in sleep duration [45]. Mental health symptoms also worsened to some extent during the early stages of the pandemic, although these changes subsequently diminished in most population groups [46]. These rapid changes in physical activity, sedentary behavior, sleep, and mental health increased scholarly interest in 24-hour movement behaviors and their health implications.

Although lockdown restrictions constrained face-to-face assessments and field-based data collection, researchers adapted by using online questionnaires, remote follow-ups, wearable devices, and other forms of contact-free data collection. These adaptations in data collection methods, together with the time required for data analysis, manuscript preparation, peer review, and publication, may partly explain the local publication peaks observed in 2022 and 2024.

The citation peak in 2022 is also consistent with broader changes in scientific citation activity during the pandemic. Although COVID-19-related papers accounted for only approximately 4% of papers published in 2020–2021, they attracted about 20% of the citations received by papers published during the same period [47]. Subsequent research showed that this citation advantage was substantial but relatively short-lived and was concentrated mainly among COVID-19 papers published early in the pandemic; papers entering the research domain later received a markedly weaker citation premium [48]. The citation peak observed in 2022 may therefore reflect the combined effects of an expanding pool of citable literature, concentrated scholarly attention to pandemic-related topics, and the temporal lag between publication and citation accumulation.

Accordingly, the apparent decline in citations after 2024 should be interpreted cautiously. Recent publications have had less time to accumulate citations, the 2026 data cover only the period through April, and the exceptional citation advantage associated with early COVID-19 research has gradually diminished. The observed decline may therefore not necessarily indicate a substantive reduction in research activity, scholarly interest, or the academic influence of this field.

As shown in Figure 3A,B, the same five countries ranked among the leading contributors in terms of both publication output and citation impact, although their relative positions differed. The United States, China, Australia, the United Kingdom, and Canada ranked first to fifth in publication output, whereas Australia recorded the highest number of citations, followed by the United States, China, Canada, and the United Kingdom. This difference indicates that the academic influence of a country in this field is determined not only by the volume of publications but also by the timing, methodological contribution, and subsequent use of its research. A previous bibliometric analysis of studies applying the 24-hour movement guidelines in children and adolescents identified North America and Europe as the principal study locations and reported that Canadian research teams had the greatest research impact [43]. In comparison, the stronger representation of China and Australia in the present analysis suggests that the geographical distribution of research has broadened as the field has expanded from guideline adherence in children and adolescents to the relationships between 24-hour movement behaviors and mental health in adults.

China’s contribution may be partly understood within its broader policy and re-search context. National initiatives, including Healthy China 2030 and the National Fitness Program (2021–2025), emphasize physical activity, reductions in sedentary behavior, and the promotion of healthy lifestyles. Although these policies do not specifically mandate research on 24-hour movement behaviors and mental health, they may have created a supportive environment for research on modifiable lifestyle behaviors. A review of China’s national physical activity and sedentary behavior policies confirmed that population-level targets, surveillance systems, and public education have become important components of the national health strategy [49]. This policy environment has coincided with a rapid increase in Chinese research on 24-hour movement behaviors since 2019, including studies examining depression and anxiety among university students [50,51]. In addition, Australia’s particularly high citation impact may partly reflect its sustained contributions to the development and contextual adaptation of integrated 24-hour movement guidelines. Released in 2020, the Canadian 24-Hour Movement Guidelines for Adults and Older Adults completed Canada’s suite of guidelines across the lifespan and subsequently served as a key reference for the development of corresponding guidelines in Australia [11]. Building on this foundation, Australia updated the evidence base and contextualized the recommendations through stakeholder consultation, culminating in the release of its national 24-hour movement guidelines for adults and older adults in 2026 [52]. Research productivity in these countries has also been supported by access to large-scale population datasets. For example, the UK Biobank combines accelerometer-derived movement data with longitudinal mental health information and has enabled prospective analyses of how reallocating sedentary time to physical activity or sleep is associated with subsequent symptoms of depression and anxiety [29].

Table 3 lists the journals with the highest publication output in this field, whereas Table 4 presents the journals in which the globally highly cited papers were published. The two tables therefore capture distinct dimensions of journal prominence. The prominent position of the *International Journal of Environmental Research and Public Health* (IJERPH) is primarily attributable to its high publication volume, whereas that of World Psychiatry is largely driven by a highly cited meta-review by Firth et al. [28]. This meta-review synthesized evidence on the roles of exercise, smoking, diet, and sleep in the prevention and treatment of mental disorders. Its substantial citation impact suggests that the broader literature on lifestyle psychiatry may constitute an important intellectual foundation for research on 24-hour movement behaviors and mental health.

The journal distribution observed in this study may reflect several considerations that shape authors’ selection of publication venues, including perceived impact, manuscript–journal fit, target readership, accessibility, and professional or social relationships [52]. Furthermore, although no formal relationship was identified between the leading journals in Table 3 and Table 4 and the highly productive institutions in Table 1 in terms of ownership, sponsorship, or official affiliation, several authors of the highly cited me-ta-review by Firth et al. [28] were affiliated with these institutions. This pattern suggests that the scholarly influence of these institutions is mediated primarily through cross-institutional collaboration and co-authorship networks rather than through formal participation in journal ownership or governance.

Figure 6A,B provide complementary perspectives on the interdisciplinary knowledge structure of this research field through direct citation and co-citation networks, respectively. According to the formal Journal Citation Reports (JCR) categories adopted in this study, *BMC Public Health* and *Frontiers in Public Health* are classified under “Public, Environmental & Occupational Health.” *Journal of Affective Disorders* is indexed in both “Clinical Neurology” and “Psychiatry,” whereas *Frontiers in Psychiatry* and *World Psychiatry* are classified under “Psychiatry.” *International Journal of Behavioral Nutrition and Physical Activity* is assigned to both “Nutrition & Dietetics” and “Physiology,” while *Mental Health and Physical Activity* spans “Psychology, Clinical” and “Psychiatry.” *Psychology of Sport and Exercise* has a broader disciplinary profile, encompassing “Psychology,” “Psychology, Applied,” “Sport Sciences,” and “Hospitality, Leisure, Sport & Tourism.” In addition, *PLoS One* and *Scientific Reports* are classified under “Multidisciplinary Sciences”; *BMC Medicine* and *Journal of Clinical Medicine* under “Medicine, General & In-ternal”; and *Healthcare* under both “Health Care Sciences & Services” and “Health Policy & Services.” However, Clarivate discontinued coverage of the *International Journal of Environmental Research and Public Health* (IJERPH) in the Web of Science Core Collection, including SCIE and SSCI, in 2023. Consequently, IJERPH no longer receives current JCR category assignments or an Impact Factor (IF). The distribution of these journals across JCR categories also underscores the interdisciplinary nature of research on 24-hour movement behaviors and mental health, which draws on public health, psychiatry, nutrition, physiology, psychology, sport science, clinical medicine, and multi-disciplinary science.

Within the direct citation network, IJERPH and *Journal of Affective Disorders* emerged as prominent cited sources, whereas articles published in *BMC Public Health* and *Inter-national Journal of Behavioral Nutrition and Physical Activity* exhibited comparatively high levels of outgoing citation activity. The co-citation network provides a complementary view of the intellectual organization of the field. Co-citation refers to the frequency with which two documents are cited together by subsequent publications and therefore reflects their intellectual or conceptual relatedness rather than the existence of a direct citation relationship between them [53]. The central cluster of the co-citation network included journals classified under “Public, Environmental & Occupational Health,” “Psychiatry,” and “Medicine, General & Internal.” This pattern indicates that population-level public health evidence, psychiatric research, and the broader clinical literature jointly constitute the principal intellectual foundation of the field.

Furthermore, the co-citation ties linking the central sources to journals in nutrition, physiology, clinical psychology, and sport science are also consistent with the increasingly interdisciplinary orientation of lifestyle psychiatry. Lifestyle psychiatry examines the role of physical activity, diet, sleep, stress management, and other modifiable health behaviors in the prevention and management of mental disorders [54]. Recent guidance from the European Psychiatric Association, together with high-level evidence syntheses, supports the integration of physical activity and multidomain lifestyle interventions into mental health care, particularly as adjuncts to established treatments. Nevertheless, the strength of the available evidence varies across study populations, intervention modalities, and outcome measures [55,56]. Overall, Figure 6A,B indicate that the field does not rely predominantly on the literature of any single discipline. Instead, its knowledge base reflects the integration of public health epidemiology, clinical and psychiatric research, and lifestyle-oriented prevention and intervention studies. The presence of multidisciplinary journals, such as PLoS ONE and Scientific Reports, further suggests that research in this field is disseminated across disciplinary boundaries through broad-scope publishing platforms.

### 4.2. Research Hotspots and Paradigm Shifts

A critical highlight of this study is the identification of a significant paradigm shift within the research themes, reflecting the changing global health landscape. Initially, as demonstrated by the keyword co-occurrence network, earlier literature heavily emphasized traditional epidemiological risk factors (e.g., “diet”, “smoking” )and their broad associations with macro-lifestyle behaviors. With the development of the 24-hour movement behaviors framework and guidelines, physical activity, sedentary behavior and sleep were not independent exposures. The keyword analysis identified ‘compositional data analysis’ (CoDA) and ‘isotemporal substitution’ as emerging terms. The methodological evolution is further reinforced by the growing application of machine learning. Simultaneously, measurement-related keywords including “ecological momentary assessment,” “accelerometer,” and “actigraphy” have shown sustained prominence in recent years, indicating the field’s increasing reliance on objective and continuous monitoring technologies rather than traditional self-reported questionnaires. Recent empirical studies exemplify the convergence of these methodological and conceptual advances. For instance, Badon et al. used wrist-worn ActiGraph wGT3X-BT accelerometers to quantify waking movement behaviors among pregnant individuals and applied compositional isotemporal substitution models to examine behavioral reallocations. Their findings demonstrated that theoretically reallocating as little as 10 min from sleep, sedentary behavior, or light physical activity toward moderate-to-vigorous physical activity (MVPA) was associated with a 20–25% lower risk of large-for-gestational-age birthweight. Importantly, the magnitude of benefit remained similar regardless of which behavior was displaced, suggesting that the critical determinant of health benefit lies in the integrated behavioral trade-off favoring MVPA rather than in the isolated reduction in any single behavior [57].

This integrated 24-hour movement behavior framework has also been increasingly extended into the mental health domain. Recent compositional data analysis studies suggest that reallocations among physical activity, sedentary behavior, and sleep are differentially associated with depressive symptoms and other mental health outcomes [58,59]. Importantly, isotemporal substitution findings indicate that these relationships may be asymmetrical, such that reductions in moderate-to-vigorous physical activity may exert disproportionately adverse mental health effects compared with the corresponding benefits gained through reverse behavioral substitutions. These findings further support the growing recognition that movement behaviors should not be evaluated in isolation, but rather as synergistic and interdependent components within the integrated 24-hour behavioral composition.

Another prominent thematic transition identified in the overlay visualization is the increasing integration of movement behavior research with aging and neurocognitive health. Keywords such as “older adults,” “dementia,” “cognitive function,” and “Alzheimer’s disease” have become increasingly central within the network, indicating rapidly growing interest in the role of movement behaviors in brain aging and neurodegenerative disease prevention. This trend likely reflects both demographic aging and the recognition that sleep disruption, prolonged sedentary behavior, and physical inactivity may exert cumulative effects on long-term cognitive trajectories. A recent study further suggests that interventions based on short bouts of accumulated physical activity aimed at interrupting prolonged sedentary time may represent feasible behavioral strategies for older adults, with potential benefits for reducing cognitive risk and promoting healthy aging [60].

Alongside physical activity and sedentary behavior, our findings reveal an equally significant evolution in how sleep is understood within the 24-hour framework. Traditional assessments centered on “sleep quality”, “disturbances”, and “daytime sleepiness”. Although concepts such as sleep regularity and circadian stability were not yet prominently represented within the current keyword structure, emerging evidence suggests that irregular sleep–wake patterns are significantly associated with an elevated risk of depression and anxiety, even when total sleep duration meets guidelines [61], an association that appears stronger in women [62] and is linked to lower health-related quality of life in middle-aged to older adults [63]. These developments indicate a potential future research direction integrating sleep timing, behavioral regularity, and circadian health within the broader 24-hour movement paradigm.

Finally, it is important to highlight that study eligibility was based on thematic relevance rather than specific diagnoses or exposures. Consequently, the included literature spanned diverse clinical and behavioral contexts. Neurodegenerative disorders, chronic physical or mental illnesses, problematic smartphone, social media, Internet, or gaming use, and psychoactive substance use were neither prerequisites for inclusion nor grounds for exclusion in their own right. Studies involving these characteristics were included if they examined associations between 24-hour movement behaviors and mental health in adults. This distinction is relevant because problematic smartphone, Internet, and gaming use has been linked to sleep disturbance and symptoms of depression and anxiety [64,65,66], while excessive or problematic social media use has been associated with adverse mental health outcomes in adults [67]. Psychoactive substance use may affect sleep duration, continuity, and architecture; however, these alterations vary across substances, and some may persist into abstinence [68]. Although the present bibliometric analysis did not quantify these effects, differences in how the primary studies defined, measured, or analytically accounted for these clinical and behavioral characteristics may have contributed to between-study heterogeneity and should be considered when interpreting the identified thematic patterns.

## 5. Limitations

However, several limitations of the present analysis warrant acknowledgment. First, the published papers were restricted to the Web of Science Core Collection database, which might overlook relevant literature from other sources, including Scopus, PubMed, Microsoft Academic, and Google Scholar. Second, studies published in languages other than English were excluded. This may have introduced language and geographic biases, potentially underrepresenting contributions from non-English-speaking or lower-income country research communities. Third, the apparent post-2024 citation decline may be a methodological artifact of indexing latency rather than a substantive trend. Fourth, bibliometric co-occurrence analyses cannot establish causal or directional relationships between research themes. Fifth, citation metrics may be affected by self-citations or citation clubs, neither of which was assessed separately. Sixth, no sensitivity analyses examined the robustness of the findings to alternative search terms or analytical thresholds. Therefore, future studies should expand database coverage, account for self-citations, test the robustness of findings using alternative search strategies and analytical thresholds, employ longitudinal network analyses to track paradigm transitions in real time, and prioritize cross-cultural validation of 24-hour movement interventions in underrepresented populations.

## 6. Implications

Although increasing attention has been directed toward the role of 24-hour movement behaviors in cognitive function and healthy aging, their implications for vulnerable populations, particularly women with perinatal depression, remain insufficiently explored. The integrated 24-hour movement framework, which considers physical activity, sedentary behavior, and sleep as interdependent behaviors within a finite daily cycle, may provide a more comprehensive perspective for future mental health interventions. Continued progress in this field will require closer integration across psychiatry, behavioral science, public health, and digital health research, reflecting a broader shift from descriptive epidemiology toward more clinically relevant and scalable behavioral intervention approaches.

## 7. Conclusions

This study investigated the knowledge atlas of literature on 24-hour movement behaviors (physical activity, sedentary behavior, and sleep) from the view of bibliometrics. Thematic evolution analysis revealed a shift from traditional epidemiological perspectives centred on broad lifestyle-related risk factors toward integrated frameworks that conceptualize physical activity, sedentary behavior, and sleep as interdependent components of a unified 24-hour behavioral composition. The growing prominence of keywords related to compositional data analysis, isotemporal substitution, actigraphy, machine learning, cognitive function, dementia, and mental disorders reflects increasing integration of objective monitoring technologies, advanced analytical approaches, and neurocognitive perspectives within the field. These developments indicate a transition from generalized behavioral risk identification toward clinically relevant movement-based approaches in the context of aging, brain health, and preventive psychiatry. The United States, China, and Australia emerged as the principal contributors to the global research landscape, while influential institutions, authors, and Q1 journals collectively shaped the intellectual structure of the field. Taken together, the present findings provide a comprehensive overview of the evolving knowledge base and research frontiers of 24-hour movement behaviors and adult mental health.

## Figures and Tables

**Table 1:** The top twenty most productive institutions.

Affiliation	Country	Articles
Harvard University	USA	36
Adelaide University	Australia	35
University of California System	USA	31
University of South Australia	Australia	29
Pennsylvania Commonwealth System of Higher Education (PCSHE)	USA	28
University of London	UK	27
University of Leicester	UK	22
KU Leuven	Belgium	21
University of Queensland	Australia	21
Karolinska Institutet	Sweden	20
Harvard University Medical Affiliates	USA	18
University of Melbourne	Australia	18
University of Ottawa	Canada	18
University of Toronto	Canada	18
Deakin University	Australia	17
Queens University—Canada	Canada	17
University College London	UK	17
University of Newcastle	Australia	17
University of North Carolina	USA	17
University of North Carolina Chapel Hill	USA	16

**Table 2:** The most prolific authors and the most cited authors.

No.	Author	Articles	No.	Author	Total Citations
1	D. Dumuid	15	1	B. Stubbs	1143
2	L. Li	8	2	D. Vancampfort	921
3	A. E. Smith	7	3	J. Firth	907
4	D. W. Dunstan	6	4	J. Sarris	898
5	Y. Li	6	5	W. Marx	895
6	A. Lisinskiene	6	6	N. Veronese	894
7	D. Majauskiene	6	7	M. Eaton	889
8	M. L. Mellow	6	8	M. Solmi	889
9	T. Olds	6	9	S. Rosenbaum	823
10	A. Sarkauskiene	6	10	L. Smith	807
11	A. Skurvydas	6	11	F. B. Schuch	790
12	B. Stubbs	6	12	G. Ashdown-Franks	784
13	D. Valanciene	6	13	A. F. Carvalho	784
14	D. Vancampfort	6	14	S. Gilbody	784
15	R. Dadeliene	5	15	E. Hoare	784
16	M. Hamer	5	16	F. N. Jacka	784
17	A. F. Kramer	5	17	S. E. Jackson	784
18	Y. Liao	5	18	D. Siskind	784
/	/	/	19	S. B. Teasdale	784
/	/	/	20	J. Torous	784
/	/	/	21	R. E. Wootton	784

“/” indicates “not applicable”.

**Table 3:** The top ten most fruitful journals.

Rank	Journal	Publications	Factor (2025)	Region (JCR)
1	BMC Public Health	31	3.6	Q1
2	International Journal of Environmental Research and Public Health	18	None	None
3	Journal of Affective Disorders	16	4.9	Q1
4	PLoS One	14	2.6	Q2
5	Frontiers in Public Health	12	3.4	Q1
6	Scientific Reports	12	3.9	Q1
7	Healthcare	11	2.7	Q2
8	International Journal of Behavioral Nutrition and Physical Activity	10	5.5	Q1
9	Nutrients	9	5	Q1
10	Journal of Clinical Medicine	8	2.9	Q1

**Table 4:** The top ten most cited journals.

Rank	Journal	Total Citations	Impact Factor (2025)	Region (JCR)
1	World Psychiatry	784	65.8	Q1
2	Nature Reviews Endocrinology	474	40	Q1
3	Proceedings of the National Academy of Sciences of the United States of America	428	9.1	Q1
4	Annual Review of Public Health	234	20.7	Q1
5	Frontiers in Immunology	174	5.9	Q1
6	JAMA Psychiatry	158	17.1	Q1
7	eClinicalMedicine	145	10	Q1
8	Biology of Sport	131	5	Q1
9	The World Journal of Biological Psychiatry	111	3.8	Q1
10	BMC Medicine	105	8.3	Q1

**Table 5:** The top ten local-cited references.

Rank	Reference	DOI	Year	Local Citations
1	Wei JK, 2021, J Sci Med Sportp [26]	10.1016/j.jsams.2020.09.013	2021	17
2	Fanning J, 2017, J Behav Med [27]	10.1007/s10865-016-9788-9	2017	16
3	McGregor DE, 2018, Int J Environ Res Public Health [12]	10.3390/ijerph15081779	2018	16
4	Firth J, 2020, World Psychiatry [28]	10.1002/wps.20773	2020	16
5	Kandola AA, 2021, BMC Med [29]	10.1186/s12916-021-02007-3	2021	15
6	Mellow ML, 2022, Exp Gerontol [30]	10.1016/j.exger.2022.111698	2022	15
7	Sdel Pozo Cruz B, 2020, J Affect Disord [31]	10.1016/j.jad.2020.01.023	2020	13
8	Olds T, 2018, PLoS One [32]	10.1371/journal.pone.0199605	2018	10
9	Curtis RG, 2020, J Phys Act Health [33]	10.1123/jpah.2018-0687	2020	8
10	Cabanas-Sánchez V, 2021, Int J Behav Nutr Phys Act [16]	10.1186/s12966-021-01194-9	2021	8

**Table 6:** The top ten global-cited references.

Rank	Reference	DOI	Total Citations
1	Firth J, 2020, World Psychiatry [28]	10.1002/wps.20773	784
2	Chaput JP, 2023, Nat Rev Endocrinol [34]	10.1038/s41574-022-00747-7	474
3	Giuntella O, 2021, Proc Natl Acad Sci USA [35]	10.1073/pnas.2016632118	428
4	Dogra S, 2022, Annu Rev Public Health [36]	10.1146/annurev-publhealth-052620-091107	234
5	You YW, 2023, Front Immunol [37]	10.3389/fimmu.2022.1080782	174
6	Merikangas KR, 2019, JAMA Psychiatry [38]	10.1001/jamapsychiatry.2018.3546	158
7	Cao Z, 2022, EClinicalMedicine [39]	10.1016/j.eclinm.2022.101424	145
8	Trabelsi K, 2021, Biol Sport [40]	10.5114/biolsport.2021.101605	131
9	Marx W, 2023, World J Biol Psychiatry [41]	10.1080/15622975.2022.2112074	111
10	Sarris J, 2020, BMC Med [42]	10.1186/s12916-020-01813-5	105

## Data Availability

The bibliographic records analyzed in this study were obtained from the Web of Science Core Collection under institutional subscription and are subject to Clarivate’s licensing conditions. The processed data and analysis code are available from the corresponding author upon reasonable request. The complete search strategy and completed PRISMA 2020 checklist are provided in the Supplementary Materials. A separate data extraction form was not used because the bibliographic metadata were exported directly from the Web of Science Core Collection.

## Supplementary Materials

The following supporting information can be downloaded at: https://www.mdpi.com/article/10.3390/healthcare14213576/s1.
