# Supplementary material for: Twenty-Four-Hour Movement Behaviors and Adult Mental Health: A Bibliometric Analysis of Global Trends, Research Hotspots, and Emerging Directions (2016–2026)
Source: Healthcare (Basel). 2026 Oct 22;14(21):3576. doi: 10.3390/healthcare14213576 (PMC13762503; doi:10.3390/healthcare14213576)
Supplement: Supplementary file 1 [file healthcare-14-03576-s001.zip › healthcare-4584701-supplementary.pdf]

Supplement File S1

#	Search Query	Results
1	"sleep" (Topic) OR "sleep patterns" (Topic) OR "sleep habits" (Topic) OR "sleeping" (Topic) Editions: WOS.SCI,WOS.SSCI	350330
2	"sedentary behavior" (Topic) OR "sedentary behaviour" (Topic) OR "sedentary lifestyle" (Topic) OR "sedentary time" (Topic) OR "screen time" (Topic) OR "tv viewing time" (Topic) Editions: WOS.SCI,WOS.SSCI	31386
3	"exercise" (Topic) OR "physical activity" (Topic) Editions: WOS.SCI,WOS.SSCI	703188
4	#1 AND #2 AND #3 Editions: WOS.SCI,WOS.SSCI	4094
5	(TS=((("24-hour" OR "24 hour" OR "24h" OR "twenty-four hour" OR "whole-day" OR "whole day" OR "daily" OR "24-h" OR "24-h day") AND ("movement* behavior*" OR "movement* behaviour*" OR "physical behavior*" OR "physical behaviour*" OR "time-use" OR "time use" OR "time-use behavior*" OR "time-use behaviour*" OR "compositional data" OR "compositional analys*" OR "composition* data" OR "composition* analys*" OR "compositional approach*" OR "compositional model*")) OR ("physical activity" AND "sleep" AND "sedentary"))) OR (((((((TS=("movement behaviors")) OR TS=("movement behaviours")) OR TS=("physical behaviours")) OR TS=("time use behaviours")) OR TS=("24-hour cycle active")) OR TS=("24 hour movement guidelines")) OR TS=("24 hour movement behaviours")) OR TS=("24 hour time use") Editions: WOS.SCI,WOS.SSCI	7799
6	#4 OR #5 Editions: WOS.SCI,WOS.SSCI	8634
7	TS=((("mental health" OR psychosocial OR psychological OR wellbeing OR well-being OR "social health" OR "social emotional") OR (depress* OR anxiety OR stress OR emotion* OR somatic) OR ("self-esteem" OR "self-perception" OR "self-concept" OR "positive affect" OR "negative affect" OR "life satisfaction" OR "satisfaction with life") OR (cognitive OR "executive function*" OR memory OR inhibition OR attention OR concentrat* OR "brain function*" OR intelligence OR "problem solv*" OR perceptual))) Editions: WOS.SCI,WOS.SSCI	12194173
8	#6 AND #7 Editions: WOS.SCI,WOS.SSCI	3376
9	TS=(((child* OR toddler* OR preschool* OR "early year*") OR (adolescen* OR teen* OR youth* OR "young people" OR "young person") OR (boy* OR girl*) OR (student* OR "school-age*" OR "elementary school*" OR "primary school*" OR "secondary school*" OR "high school*") OR (pediatric*))) Editions: WOS.SCI,WOS.SSCI	3676177

10	(#8) NOT #9Editions: WOS.SCI,WOS.SSCI	1829
11	(#8) NOT #9 and 2026 or 2025 or 2024 or 2023 or 2022 or 2021 or 2020 or 2019 or 2018 or 2017 or 2016 (Publication Years) Editions: WOS.SCI,WOS.SSCI	1435
12	(#8) NOT #9 and 2026 or 2025 or 2024 or 2023 or 2022 or 2021 or 2020 or 2019 or 2018 or 2017 or 2016 (Publication Years) and English (Languages) Editions: WOS.SCI,WOS.SSCI	1423
13	(#8) NOT #9 and 2026 or 2025 or 2024 or 2023 or 2022 or 2021 or 2020 or 2019 or 2018 or 2017 or 2016 (Publication Years) and English (Languages) and Early Access or Meeting Abstract or Editorial Material or Proceeding Paper or Correction or Letter (Exclude – Document Types) Editions: WOS.SCI,WOS.SSCI	1372
	Exclude topic-unrelated literature	845
	In total	527
